# Development of a novel method for rapid cloning of shRNA vectors, which successfully knocked down CD44 in mesenchymal triple-negative breast cancer cells

**DOI:** 10.1186/s40880-018-0327-7

**Published:** 2018-09-24

**Authors:** Lei Zhou, Dandan Sheng, Qiaodan Deng, Dong Wang, Suling Liu

**Affiliations:** 1 The CAS Key Laboratory of Innate Immunity and Chronic Disease, School of Life Sciences and Medical Center, University of Science & Technology of China, Hefei, 230027 Anhui P. R. China; 2 Fudan University Shanghai Cancer Center & Institutes of Biomedical Sciences, Shanghai Medical College, Key Laboratory of Breast Cancer in Shanghai, Innovation Center for Cell Signaling Network, Cancer Institutes, Department of Breast Surgery, Fudan University, Shanghai, 200032 P. R. China

**Dear Editor,**

Since the discovery of short hairpin RNA (shRNA) vector-mediated RNA interference (RNAi), this technology has been widely used in cancer research for its specificity, potency, and convenience. However, researchers may find it costly to purchase commercial vectors from bio-companies or time- and labor-consuming to construct their own shRNA vectors using traditional method by inserting annealed duplex into digested vectors. Despite intensive efforts to accelerate shRNA vector cloning in laboratories, the development of a reliable, rapid, convenient, and cost-effective method is still in great demand. To this end, we developed a novel method named SuperSH (Super rapid cloning of shRNA vector) for the effective and rapid construction of shRNA-expressing vectors based on high-performance DNA polymerase and seamless cloning technique [1] (Additional file 1: Figure S1a; the detailed methods can be found in Additional file 1). In our SuperSH method, the shRNA sequences are introduced into the vector via a pair of polymerase chain reaction (PCR) primers rather than via annealed duplex. In detail, the 3′ ends of the primers are designed to bind the template to initiate a PCR to amplify the vector backbone, and the 5′ portions are designed to introduce the sequences of interest as well as to form a short homologous arm for subsequent recombination via seamless cloning [1]. After the seamless recombination reaction, the seamed vector is transformed into competent *E. coli* using a quick transformation protocol that takes only 5 min [2] (Additional file 1: Figure S1). It’s important to note that the SuperSH method requires a linearized vector template to achieve effective and rapid cloning, skipping the need for purification steps throughout the cloning procedure. For this aim, we created an intermediate vector pSuperSH-MX by introducing restriction enzyme sites for two non-isocaudomers, *Mlu*I and *Xba*I, to allow complete linearization of the template plasmid and to avoid self-ligation in subsequent cloning procedures as well.

Our SuperSH method outperforms traditional shRNA cloning method based on annealed complementary oligonucleotide duplex in the following aspects (Additional file 1: Figure S1b). Firstly, the SuperSH method requires only three steps, namely a low-cycle number PCR to amplify the vector backbone, a recombination reaction to seam the vector, and a quick transformation to replicate the vectors in *E. coli*, which altogether greatly saves researchers’ hands-on work. Secondly, the above procedures also allow users to complete shRNA vector cloning in as short as 30 min. Thirdly, SuperSH uses much shorter oligonucleotides in the application of shRNA cloning, as seamless cloning requires only a short homologous arm (10–15 nucleotides) to cyclize the PCR product, and thus there is no need to synthesize the full length of shRNA sequence (Additional file 1: Figure S2). In addition, we observed this novel method to be much more reliable and accurate than traditional cloning methods, by achieving an overall success rate higher than 95% in our work. Furthermore, SuperSH is a versatile method which allows researchers to construct various shRNA vector formats, such as 21-mer-, 29-mer-, and miR-N-shRNA vectors [3] (Additional file 1: Figure S2).

To manifest the efficiency of the SuperSH method in shRNA vector cloning, we constructed five 21-mer shRNA vectors targeting CD44 along with viral backbone vectors to package lentivirus (Additional file 1: Table S1). We are interested in CD44 because it has been recently reported to play a vital role in the invasion of breast cancer by promoting the formation of invadopodia and acting as a scaffold for matrix metalloproteinases [4] and has been regarded as an important gene for promoting metastasis [5]. Meanwhile, it is well known that triple-negative breast cancer (TNBC) represents the most lethal subtype of breast cancer due to its resistance to traditional chemotherapy and radiotherapy, as well as aberrantly high ability of invasion and metastasis. Thus, we wondered if there was an association between CD44 expression and TNBC malignancy. To address this question, we analyzed the Affymetrix-chip data and RNA-seq data of various breast cancer cell lines from the Broad Institute Cancer Cell Line Encyclopedia (CCLE) [6] and found that CD44 was expressed at a significantly higher level in TNBC cell lines than in the non-TNBC cell lines (Additional file 1: Figure S3a, b). However, we also noticed that some TNBC cell lines showed low CD44 expression, indicating a heterogeneous composition of the TNBC category. Consistent with this hypothesis, Lehmann et al. [7] have reported that TNBC can be divided into seven categories according to the expression profiles of breast cancers and breast cancer cell lines. After reanalyzing the CCLE data according to Lehmann’s classification, our results explicitly revealed that basal [basal-like1 (BL1) and basal-like2 (BL2)] and mesenchymal [mesenchymal-like (M) and mesenchymal stem-like (MSL)] subcategories of TNBC, rather than the other three subtypes immunomodulatory (IM), luminal androgen receptor (LAR), and unclassified (UNS) (collectively referred to as Others hereafter) [7], exhibited a significantly higher level of CD44 mRNA (Additional file 1: Figure S3c, d). The above results demonstrated that CD44 mRNA was significantly overexpressed in basal and mesenchymal subcategories of TNBC. We then determined the protein level of CD44 in breast cancer cell lines, and our results intriguingly indicated that CD44 protein seemed exclusively overexpressed in the mesenchymal subcategory (Additional file 1: Figure S3e). These results suggest a potential association between high CD44 expression and invasion, and even subsequent metastasis in mesenchymal TNBC, as it is well known that the mesenchymal state of breast cancer contributes to invasion and metastasis [8]. Interestingly, we also noted that the mesenchymal TNBC cell lines were enriched for cancer stem cells indicated by CD24^−^/CD44^+^ [8] (Additional file 1: Figure S4a), where the CD24^−^/CD44^+^ population accounted for almost 100% in the non-targeting (NT) control of all three cell lines.

To further decipher the role of CD44 in mesenchymal TNBC cell lines, we stably knocked down CD44 in three mesenchymal TNBC cell lines (SUM159, MDA-MB-436, and MDA-MB-231) using shRNA vectors constructed by the above described SuperSH method. After selection for stable cell lines, we determined the knockdown efficiency of the shRNA vectors in the TNBC cell lines. Western blotting showed that the shRNA clones sh4 and sh5 achieved the best knockdown efficiency in all three cell lines (Fig. 1a–c), which was also confirmed by flow cytometry with fluorescence-conjugated antibody for CD44 (Additional file 1: Figure S4a). Therefore, we used these two shRNAs in subsequent studies to assess the role of CD44 in cell proliferation, colony formation, and invasion. Our results showed that deletion of CD44 significantly suppressed cell proliferation (Fig. 1d–f), colony formation (Fig. 1g, h) and invasion ability (Fig. 1i, j) of mesenchymal TNBC cell lines SUM159, MDA-MB-436, and MDA-MB-231, indicating a vital role of CD44 in promoting the proliferation and invasion of mesenchymal TNBC cell lines, consistent with previous reports in other cancers [9].

**Fig. 1:**
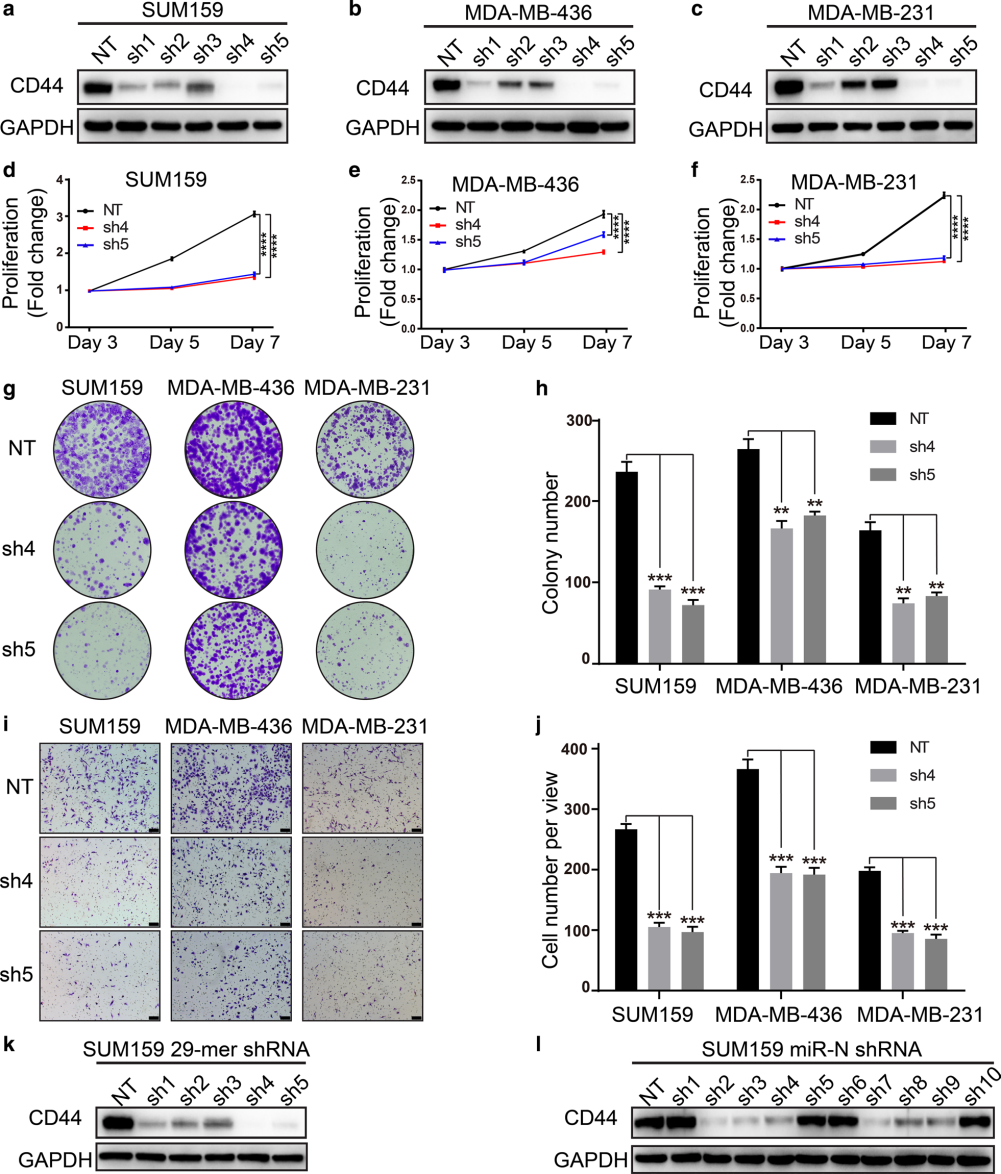
The knocking down of CD44 markedly suppresses the proliferation and invasion of mesenchymal TNBC cell lines. **a**–**c** The efficiency of shRNA vectors in knocking down CD44 was determined in stable cell lines at the protein level. **d**–**f** MTT assay was performed in stable cell lines to determine the effect of CD44 knockdown on cell proliferation. The OD490 value was read at three indicated time points and the values were normalized to that of day 3. The results are shown as mean ± SEM. ****P < 0.0001; this experiment was independently performed 3 times. **g**, **h** Colony formation assay was performed to assess the effect of CD44 knockdown on colony formation ability. **i**, **j** Transwell assay was carried out to decipher the impact of CD44 knockdown on invasion. A representative picture of three independent experiments is shown for each cell line and the related statistic results are shown as mean ± SEM. **P < 0.01; ***P < 0.001; this experiment was independently performed 3 times. Scale: The black rectangular box at the right bottom corners of figure i represents the 100 µm scale. **k**, **l** Western blotting was carried out in SUM159 cells with CD44 stably interfered with 29-mer shRNA and miR-N shRNA, respectively. *TNBC* triple-negative breast cancer, *shRNA* short hairpin RNA, *MTT assay* 3-(4,5-dimethylthiazol-2-yl)-2,5-diphenyltetrazolium bromide assay, *OD490* optical density at 490 nm, *SEM* standard error of the mean, *NT* non-targeting, *GAPDH* glyceraldehyde-3-phosphate dehydrogenase

To demonstrate the versatility of the SuperSH method in cloning various forms of shRNA vectors, we also constructed shRNA vectors targeting CD44 in two other forms, namely 29-mer shRNA and miR-N shRNA [3] (Additional file 1: Table S1) and determined their knockdown efficiency by using both Western blotting (Fig. 1k, l) and flow cytometry (Additional file 1: Figure S4b, c). In our results, both shRNA formats achieved excellent knockdown efficiency with their best-performing clones, which were comparable to that of the best 21-mer shRNA vectors described above. To provide additional support to this novel shRNA vector construction technique, we also knocked down several other genes, such as vimentin (VIM) and glyceraldehyde-3-phosphate dehydrogenase (GAPDH) and achieved robust knocking-down effects in breast cancer cell line SUM159 (Additional file 1: Figure S5).

In summary, we developed a rapid, reproducible, and cost-effective method for shRNA vector cloning, which would evidently facilitate the shRNA cloning work for researchers, especially if they are not able to access commercial shRNA libraries. Using this method, we have successfully constructed several forms of shRNA vectors targeting one of the widely accepted breast cancer stem cell marker CD44 whose expression has been previously implied to promote metastasis in breast cancer. To better understand the roles of CD44 in breast cancer, we characterized the CD44 expression pattern in non-TNBC and TNBC and found that CD44 was significantly overexpressed in the mesenchymal subcategory of TNBC cell lines at both mRNA and protein level (Additional file 1: Figure S3). Epithelial-mesenchymal transition (EMT) state has been demonstrated to be involved in breast cancer invasion and subsequent metastasis [10]. Hence, our analysis of the CD44 expression pattern implies a vital role in these processes. Consistent with this notion, when we stably knocked down CD44 in mesenchymal TNBC cell lines, the invasion ability of all three tested mesenchymal breast cancer cell lines, SUM159, MDA-MB-436, and MDA-MB-231, were markedly inhibited, suggesting an essential function of CD44 in mesenchymal TNBC invasion. Our results may, to some extent, put forward the hope of targeting CD44 in mesenchymal TNBC to inhibit its proliferation, invasion, and metastasis and to obtain a better prognosis for breast cancer.

## Additional file

**Additional file 1.** Additional materials.

## Abbreviations

BL1: basal-like 1
BL2: basal-like 2
CCLE: Broad Institute Cancer Cell Line Encyclopedia
EMT: epithelial–mesenchymal transition
GAPDH: glyceraldehyde-3-phosphate dehydrogenase
IM: immunomodulatory
LAR: luminal androgen receptor
M: mesenchymal-like
MSL: mesenchymal stem-like
MTT assay: 3-(4,5-dimethylthiazol-2-yl)-2,5-diphenyltetrazolium bromide assay
NT: non-targeting
PCR: polymerase chain reaction
RNAi: RNA interference
shRNA: short hairpin RNA
SuperSH: super rapid cloning of shRNA vector
