# Supplementary material for: Development of a novel method for rapid cloning of shRNA vectors, which successfully knocked down CD44 in mesenchymal triple-negative breast cancer cells
Source: Cancer Commun (Lond). 2018 Sep 24;38:57. doi: 10.1186/s40880-018-0327-7 (PMC6154939; doi:10.1186/s40880-018-0327-7)
Supplement: Supplementary file 1 — Additional file 1. Additional materials. [file 40880_2018_327_MOESM1_ESM.docx]

**Additional files**

**Additional Figures and Tables:**

**
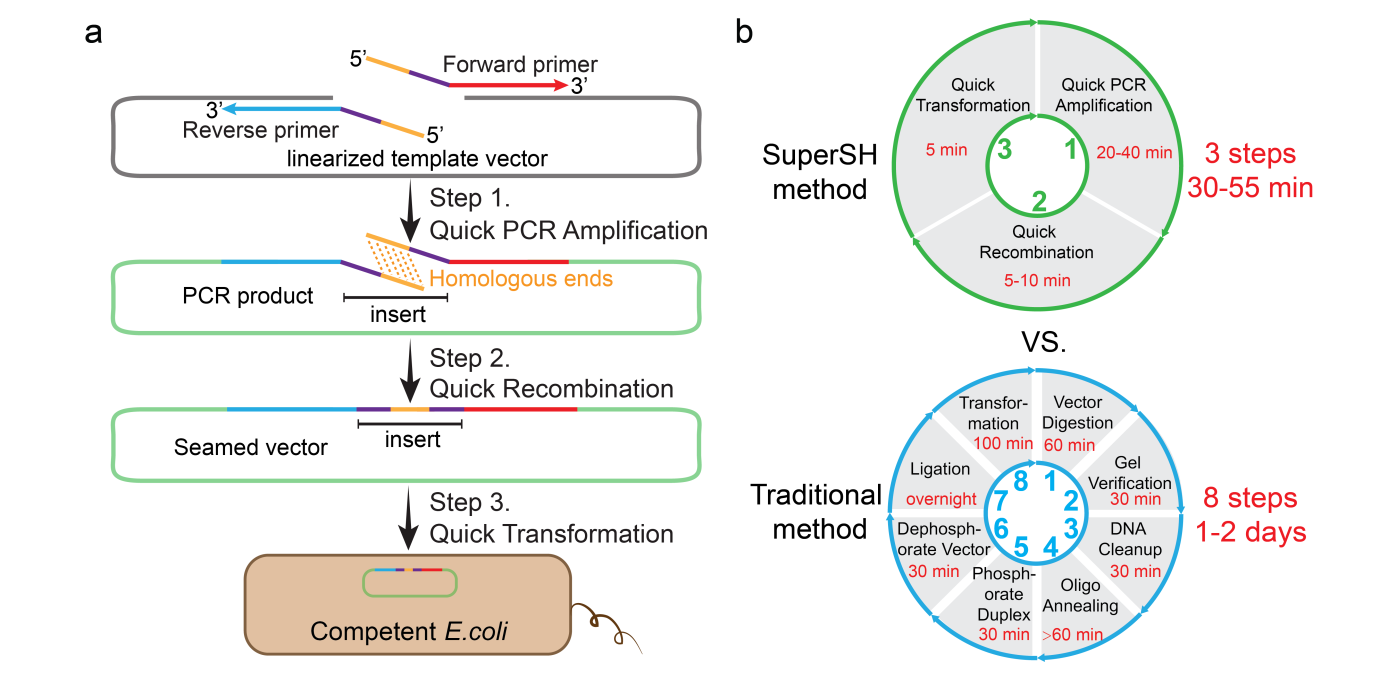
**

**Figure S1. SuperSH method allows the super rapid cloning of shRNA vectors.** **a.** A schematic representation of the SuperSH cloning procedures. Linearized template vector is used as the template for PCR with a pair of primers containing essential sequences for subsequent cloning in their 5' ends. The PCR product is directly subjected to recombination reaction to produce a seamed vector, which is subsequently transformed into bacteria following a quick protocol. **b** Comparison between the SuperSH method and the traditional shRNA cloning method in terms of time and steps. The approximate time required for each step is indicated in red. Abbreviations: shRNA, short hairpin RNA; SuperSH Super rapid cloning of shRNA vector; PCR, Polymerase Chain Reaction.


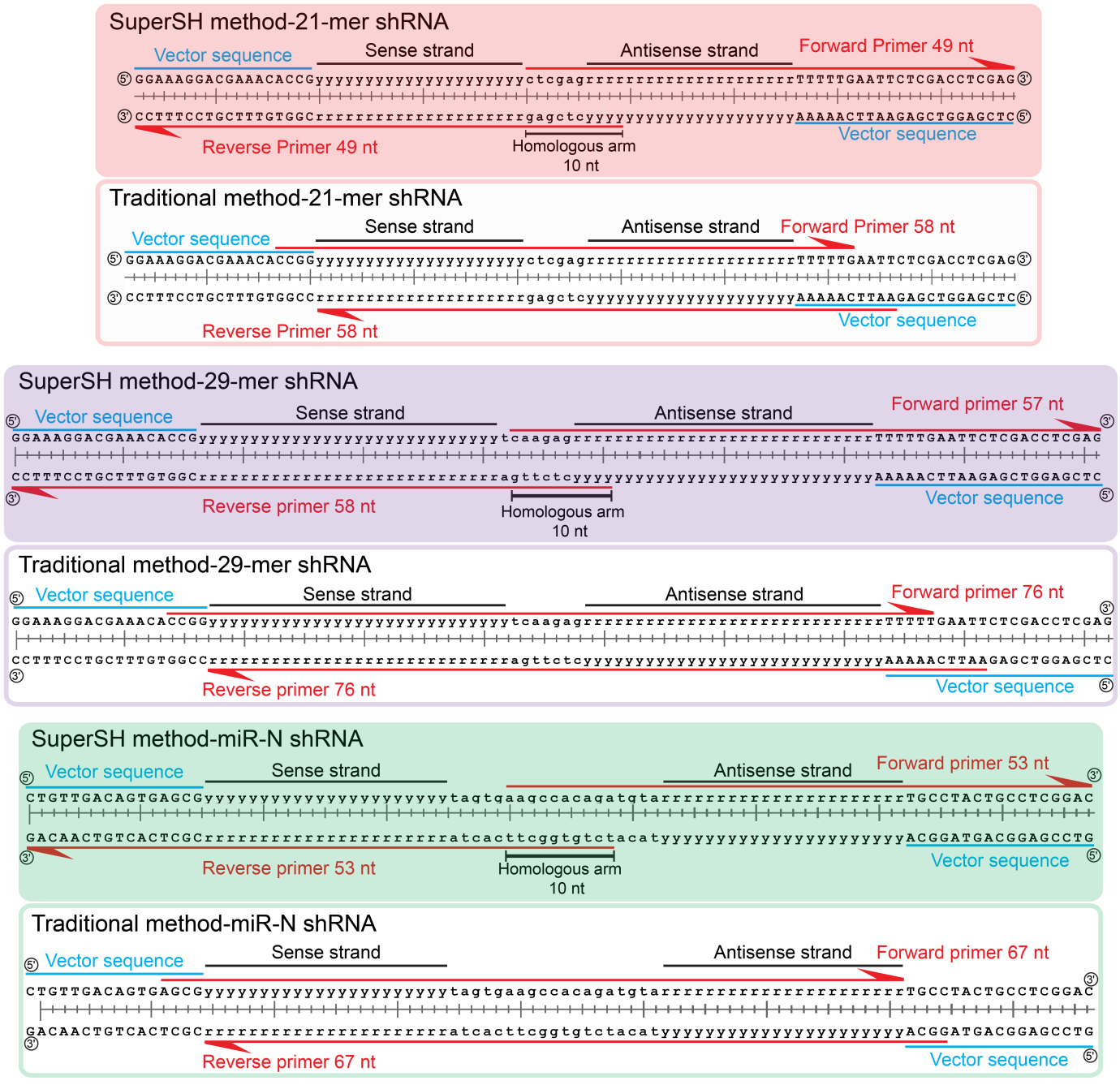


**Figure S2. The SuperSH method uses shorter primers than the traditional method in shRNA cloning.** The SuperSH method takes advantage of the seamless cloning technique and hence skipping the need to synthesize the full-length inserting sequences, which results in shorter oligonucleotides to be used. In the examples of cloning 21-mer, 29-mer, and miR-N shRNA vectors, SuperSH uses 49-nt, 57-nt, and 53-nt oligonucleotides, whereas the traditional method uses 58-nt, 76-nt, and 67-nt oligonucleotides, respectively. Sense strand (y), antisense strand (r), and vector sequences (blue line) are indicated by lines. Abbreviations: shRNA, short hairpin RNA; SuperSH Super rapid cloning of shRNA vector; nt, nucleotide.


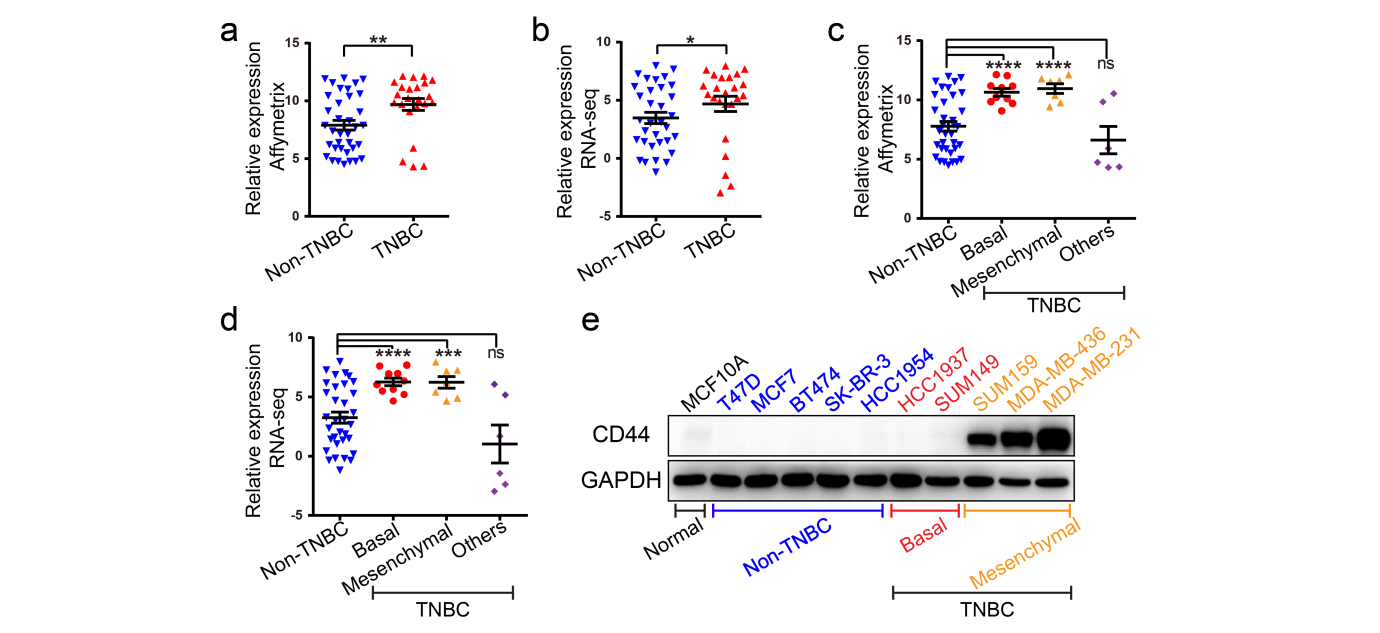


**Figure S3. CD44 is highly expressed in mesenchymal-like TNBC cell lines.** **a**-**b** CD44 expression data retrieved from CCLE (<https://portals.broadinstitute.org/ccle/about>) [[1](#_ENREF_1)] was analyzed according to Non-TNBC and TNBC categories. **c**-**d.** The above data was re-analyzed with TNBC divided into three categories, namely basal, mesenchymal and others (including IM, LAR, and UNS). *, *P* < 0.05; **, *P* < 0.01; ***, *P* < 0.001; ****, *P* < 0.0001; ns, no significance. **e.** CD44 protein expression in breast cancer cell lines was determined by Western blotting, with GAPDH as a loading control. Abbreviations: TNBC, Triple-Negative Breast Cancer; CCLE, Broad Institute Cancer Cell Line Encyclopedia; IM, Immunomodulatory; LAR, Luminal Androgen Receptor; UNS, Unclassified Subcategories.


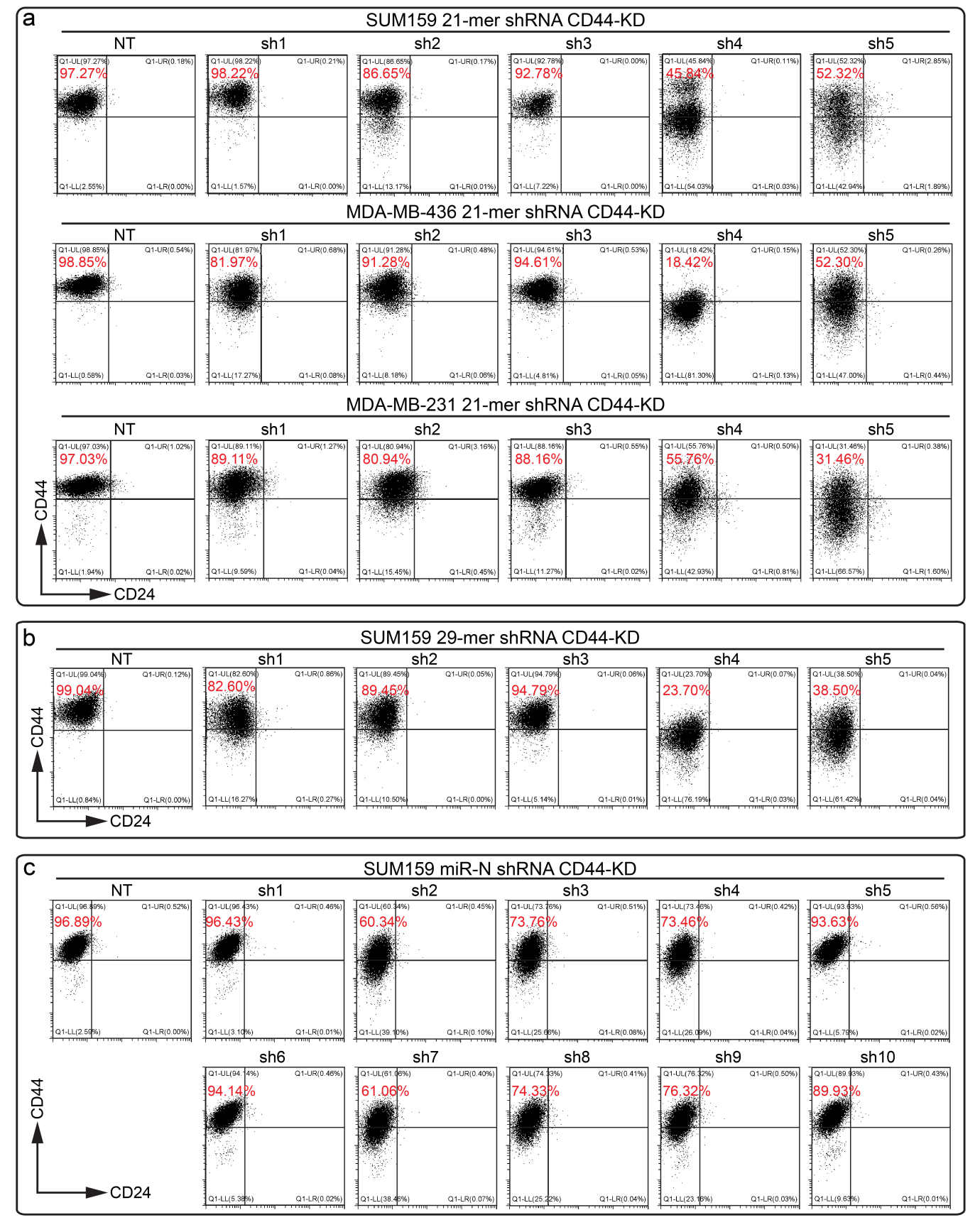


**Figure S4. The knockdown efficiency of shRNAs targeting CD44 was assessed by flow cytometry. a** CD44 knockdown with 21-mer shRNA vectors in SUM159, MDA-MB-436, and MDA-MB-231 cells was assessed with CD24 and CD44 staining. **b-c** CD44 knockdown by 29-mer and miR-N shRNA vectors in SUM159 cells was confirmed by flow cytometry. Abbreviations: shRNA, short hairpin RNA; NT, Non-Targeting; KD, Knocking Down.


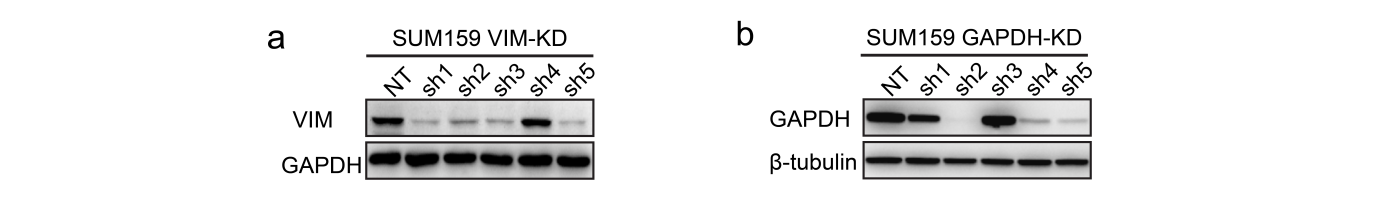


**Figure S5. VIM and GAPDH were knocked down in SUM159 cells with 21-mer shRNA vectors constructed by the SuperSH method.** The shRNA vectors targeting VIM and GAPDH were constructed, and stable cell lines were established. Western blotting was performed to determine the knockdown efficiency for (**a**) VIM and (**b**) GAPDH, with GAPDH and β-tubulin as a loading control, respectively. Abbreviations: KD, Knocking Down; VIM, Vimentin; GAPDH, Glyceraldehyde-3-phosphate Dehydrogenase; shRNA, short hairpin RNA; SuperSH, Super rapid cloning of shRNA vector.

**Table S1. Primers used in shRNA vector cloning.**

| **shRNA clone** | **Primer sequence** |
| --- | --- |
| **CD44-21-mer-NT** | Forward, 5'-CTCGAGTTGGTGCTCTTCATCTTGTTGTTTTTGAATTCTCGACCTCGAG-3' |
|  | Reverse, 5'-CCAACTCGAGTTGGTGCTCTTCATCTTGTTGCGGTGTTTCGTCCTTTCC-3' |
| **CD44-21-mer-sh1** | Forward, 5'-CTCGAGTTCTCCTTTCTGGACATAGCGTTTTTGAATTCTCGACCTCGAG-3' |
|  | Reverse, 5'-AGAACTCGAGTTCTCCTTTCTGGACATAGCGCGGTGTTTCGTCCTTTCC-3' |
| **CD44-21-mer-sh2** | Forward, 5'-CTCGAGAATATGTGTCATACTGGGAGGTTTTTGAATTCTCGACCTCGAG-3' |
|  | Reverse, 5'-TATTCTCGAGAATATGTGTCATACTGGGAGGCGGTGTTTCGTCCTTTCC-3' |
| **CD44-21-mer-sh3** | Forward, 5'-CTCGAGTTTGGAAATCACTAATAGGGCTTTTTGAATTCTCGACCTCGAG-3' |
|  | Reverse, 5'-AAACTCGAGTTTGGAAATCACTAATAGGGCGGTGTTTCGTCCTTTCC-3' |
| **CD44-21-mer-sh4** | Forward, 5'-CTCGAGTAATGGTTATGTTTCCAACGGTTTTTGAATTCTCGACCTCGAG-3' |
|  | Reverse, 5'-ATTACTCGAGTAATGGTTATGTTTCCAACGGCGGTGTTTCGTCCTTTCC-3' |
| **CD44-21-mer-sh5** | Forward, 5'-CTCGAGAATAGTTATGGTAATTGGTCCTTTTTGAATTCTCGACCTCGAG-3' |
|  | Reverse, 5'-TATTCTCGAGAATAGTTATGGTAATTGGTCGGTGTTTCGTCCTTTCC-3' |
| **CD44-29-mer-NT** | Forward, 5'-CAAGAGAGTACTATCTGAGTTAGCTCTGGTAGTGCTTTTTGAATTCTCGACCTCGAG-3' |
|  | Reverse, 5'-TACTCTCTTGAAGTACTATCTGAGTTAGCTCTGGTAGTGCGGTGTTTCGTCCTTTCC-3' |
| **CD44-29-mer-sh1** | Forward, 5'-CAAGAGGTTCTGTATTCTCCTTTCTGGACATAGCGTTTTTGAATTCTCGACCTCGAG-3' |
|  | Reverse, 5'-GAACCTCTTGAGTTCTGTATTCTCCTTTCTGGACATAGCGCGGTGTTTCGTCCTTTCC-3' |
| **CD44-29-mer-sh2** | Forward, 5'-CAAGAGATTGAAGCAATATGTGTCATACTGGGAGGTTTTTGAATTCTCGACCTCGAG-3' |
|  | Reverse, 5'-CAATCTCTTGAATTGAAGCAATATGTGTCATACTGGGAGGCGGTGTTTCGTCCTTTCC-3' |
| **CD44-29-mer-sh3** | Forward, 5'-CAAGAGATATTGTTTTTGGAAATCACTAATAGGGCTTTTTGAATTCTCGACCTCGAG-3' |
|  | Reverse, 5'-ATATCTCTTGAATATTGTTTTTGGAAATCACTAATAGGGCGGTGTTTCGTCCTTTCC-3' |
| **CD44-29-mer-sh4** | Forward, 5'-CAAGAGGCTCCCTGTAATGGTTATGTTTCCAACGGTTTTTGAATTCTCGACCTCGAG-3' |
|  | Reverse, 5'-GAGCCTCTTGAGCTCCCTGTAATGGTTATGTTTCCAACGGCGGTGTTTCGTCCTTTCC-3' |
| **CD44-29-mer-sh5** | Forward, 5'-CAAGAGCGGTTAACAATAGTTATGGTAATTGGTCCTTTTTGAATTCTCGACCTCGAG-3' |
|  | Reverse, 5'-ACCGCTCTTGACGGTTAACAATAGTTATGGTAATTGGTCCGGTGTTTCGTCCTTTCC-3' |
| **CD44-miR-N-NT** | Forward, 5'-AAGCCACAGATGTACTTACTCTCGCCCAAGCGAGAGTGCCTACTGCCTCGGAC-3' |
|  | Reverse, 5'-TCTGTGGCTTCACTACTTACTCTCGCCCAAGCGAGATCGCTCACTGTCAACAG-3' |
| **CD44-miR-N-sh1** | Forward, 5'-AAGCCACAGATGTATTTTATTCGAGGTTGAAAACAGTGCCTACTGCCTCGGAC-3' |
|  | Reverse, 5'-TCTGTGGCTTCACTATTTTATTCGAGGTTGAAAACATCGCTCACTGTCAACAG-3' |
| **CD44-miR-N-sh2** | Forward, 5'-AAGCCACAGATGTATTCGACTGTTGACTGCAATGCATGCCTACTGCCTCGGAC-3' |
|  | Reverse, 5'-TCTGTGGCTTCACTATTCGACTGTTGACTGCAATGCGCGCTCACTGTCAACAG-3' |
| **CD44-miR-N-sh3** | Forward, 5'-AAGCCACAGATGTATTCAAATCGATCTGCGCCAGGCTGCCTACTGCCTCGGAC-3' |
|  | Reverse, 5'-TCTGTGGCTTCACTATTCAAATCGATCTGCGCCAGGTCGCTCACTGTCAACAG-3' |
| **CD44-miR-N-sh4** | Forward, 5'-AAGCCACAGATGTATCAAATCCGATGCTCAGAGCTTTGCCTACTGCCTCGGAC-3' |
|  | Reverse, 5'-TCTGTGGCTTCACTATCAAATCCGATGCTCAGAGCTGCGCTCACTGTCAACAG-3' |
| **CD44-miR-N-sh5** | Forward, 5'-AAGCCACAGATGTATTAAGAAGTAGCAGCCCTCCCGTGCCTACTGCCTCGGAC-3' |
|  | Reverse, 5'-TCTGTGGCTTCACTATTAAGAAGTAGCAGCCCTCCCTCGCTCACTGTCAACAG-3' |
| **CD44-miR-N-sh6** | Forward, 5'-AAGCCACAGATGTATTTAAGAAGTAGCAGCCCTCCCTGCCTACTGCCTCGGAC-3' |
|  | Reverse, 5'-TCTGTGGCTTCACTATTTAAGAAGTAGCAGCCCTCCTCGCTCACTGTCAACAG-3' |
| **CD44-miR-N-sh7** | Forward, 5'-AAGCCACAGATGTATATATTCAAATCGATCTGCGCCTGCCTACTGCCTCGGAC-3' |
|  | Reverse, 5'-TCTGTGGCTTCACTATATATTCAAATCGATCTGCGCTCGCTCACTGTCAACAG-3' |
| **CD44-miR-N-sh8** | Forward, 5'-AAGCCACAGATGTATTGAAAGCCTTGCAGAGGTCAGTGCCTACTGCCTCGGAC-3' |
|  | Reverse, 5'-TCTGTGGCTTCACTATTGAAAGCCTTGCAGAGGTCATCGCTCACTGTCAACAG-3' |
| **CD44-miR-N-sh9** | Forward, 5'-AAGCCACAGATGTATATTGAAAGCCTTGCAGAGGTCTGCCTACTGCCTCGGAC-3' |
|  | Reverse, 5'-TCTGTGGCTTCACTATATTGAAAGCCTTGCAGAGGTTCGCTCACTGTCAACAG-3' |
| **CD44-miR-N-sh10** | Forward, 5'-AAGCCACAGATGTATTATTCGAGGTTGAAAACAGTGTGCCTACTGCCTCGGAC-3' |
|  | Reverse, 5'-TCTGTGGCTTCACTATTATTCGAGGTTGAAAACAGTTCGCTCACTGTCAACAG-3' |
| **VIM-21-mer-sh1** | Forward, 5'-CTCGAGAATAGTGTCTTGGTAGTTAGCTTTTTGAATTCTCGACCTCGAG-3' |
|  | Reverse, 5'-TATTCTCGAGAATAGTGTCTTGGTAGTTAGCGGTGTTTCGTCCTTTCC-3' |
| **VIM -21-mer-sh2** | Forward, 5'-CTCGAGAAGTTTCGTTGATAACCTGTCTTTTTGAATTCTCGACCTCGAG-3' |
|  | Reverse, 5'-ACTTCTCGAGAAGTTTCGTTGATAACCTGTCGGTGTTTCGTCCTTTCC-3' |
| **VIM -21-mer-sh3** | Forward, 5'-CTCGAGTTGAACTCGGTGTTGATGGCGTTTTTGAATTCTCGACCTCGAG-3' |
|  | Reverse, 5'-ACTTCTCGAGTTGAACTCGGTGTTGATGGCGCGGTGTTTCGTCCTTTCC-3' |
| **VIM -21-mer-sh4** | Forward, 5'-CTCGAGTTGAGTGGGTATCAACCAGAGTTTTTGAATTCTCGACCTCGAG-3' |
|  | Reverse, 5'-TCAACTCGAGTTGAGTGGGTATCAACCAGAGCGGTGTTTCGTCCTTTCC-3' |
| **VIM -21-mer-sh5** | Forward, 5'-CTCGAGATATTCTGAATCTCATCCTGCTTTTTGAATTCTCGACCTCGAG-3' |
|  | Reverse, 5'-TATTCTCGAGATATTCTGAATCTCATCCTGCGGTGTTTCGTCCTTTCC-3' |
| **GAPDH-21-mer-sh1** | Forward, 5'-CTCGAGAAGTCAGAGGAGACCACCTGGTTTTTGAATTCTCGACCTCGAG-3' |
|  | Reverse, 5'-ACTTCTCGAGAAGTCAGAGGAGACCACCTGGCGGTGTTTCGTCCTTTCC-3' |
| **GAPDH-21-mer-sh2** | Forward, 5'-CTCGAGATGTAAACCATGTAGTTGAGGTTTTTGAATTCTCGACCTCGAG-3' |
|  | Reverse, 5'-ACATCTCGAGATGTAAACCATGTAGTTGAGGCGGTGTTTCGTCCTTTCC-3' |
| **GAPDH-21-mer-sh3** | Forward, 5'-CTCGAGATCACGCCACAGTTTCCCGGATTTTTGAATTCTCGACCTCGAG-3' |
|  | Reverse, 5'-TGATCTCGAGATCACGCCACAGTTTCCCGGACGGTGTTTCGTCCTTTCC-3' |
| **GAPDH-21-mer-sh4** | Forward, 5'-CTCGAGTTGTCATACCAGGAAATGAGCTTTTTGAATTCTCGACCTCGAG-3' |
|  | Reverse, 5'-ACAACTCGAGTTGTCATACCAGGAAATGAGCGGTGTTTCGTCCTTTCC-3' |
| **GAPDH-21-mer-sh5** | Forward, 5'-CTCGAGATTGATGGCAACAATATCCACTTTTTGAATTCTCGACCTCGAG-3' |
|  | Reverse, 5'-CAATCTCGAGATTGATGGCAACAATATCCACGGTGTTTCGTCCTTTCC-3' |

**Note:** Forward and reverse primers for construction of 21-mer, 29-mer, and miR-N shRNA vectors targeting CD44, VIM, and GAPDH following the SuperSH method are listed, with guide (antisense) sequences highlighted in red. Abbreviations: shRNA, short hairpin RNA; VIM, Vimentin; GAPDH, Glyceraldehyde-3-phosphate Dehydrogenase; SuperSH, Super rapid cloning of shRNA vector.

**Methods:**

**Cell Culture**

HEK293T, MDA-MB-436, and MDA-MB-231 cells were purchased from ATCC and cultured according to the ATCC protocols. HEK293T was maintained in high glucose DMEM medium (Gibco, CA, USA) supplemented with 10% FBS (Gibco, CA, USA) and 1% penicillin-streptomycin antibiotic (Beyotime, Shanghai, China). MDA-MB-436 was cultured with Leibovitz's L-15 (Gibco, CA, USA) medium supplemented with 10% FBS (Gibco, CA, USA), 1% penicillin-streptomycin antibiotic (Beyotime, Shanghai, China), 10 μg/mL insulin (Gibco, CA, USA), and 16 μg/mL glutathione (Thermo Fisher, MA, USA). MDA-MB-231 was cultured in RPMI1640 medium (Gibco, CA, USA) supplemented with 10% FBS (Gibco, CA, USA), 1% penicillin-streptomycin antibiotic (Beyotime, Shanghai, China), and 4 μg/mL gentamicin (Sigma, LA, USA). SUM159 cell [[2](#_ENREF_2)] was purchased from Asterand (MI, USA) and cultured with Ham’s F-12 (Gibco, CA, USA) supplemented with 5% FBS (Gibco, CA, USA), 1% penicillin-streptomycin antibiotic (Beyotime, Shanghai, China), 5 μg/mL insulin (Gibco, CA, USA) 1 μg/mL hydrocortisone (Sigma, LA, USA), and 4 μg/mL gentamicin (Sigma, LA, USA). All cell lines were maintained at 37°C in an atmosphere of 5% CO2. Mycoplasma contamination was routinely detected to avoid unexpected interference to the scientific results. Abbreviations: FBS, Fetal Bovine Serum.

**Lentivirus production and lentiviral transfection**

The shRNA vectors were co-transfected with plasmids encoding gag/pol and VSVG into HEK293T packaging cells using polyethylenimine (765090-1G, Sigma, LA, USA). SUM159 cells were infected with filtered lentivirus in the presence of 1μg/mL polybrene (107689-10G, Sigma, LA, USA) and selected with 5 μg/mL puromycin (Gibco, CA, USA) for at least 7 days to establish stable cell lines. Abbreviations: gag/pol, Gag-Pol polyprotein; VSVG, Vesicular Stomatitis Virus (VSV) G-protein.

**Polymerase chain reaction (PCR)**

The high-fidelity DNA polymerase PrimeStar MasterMix used in this work was purchased from Takara (#R045A, Tokyo, Japan). The PCR protocol mainly followed the manufacturer’s instruction with mild modifications. In brief, the PCR mix with 40 ng templates in a total volume of 10 μL was denatured at 98°C for 5 s, followed by primer annealing to templates at 55°C for 5 s and elongating at 72°C for 35 s. After 9-15 cycles, the mixture was incubated at 72°C for 3 min and cooled down to 4°C for subsequent experiments.

**Seamless cloning**

Seamless cloning kit used in this study was purchased from Vazyme (C112-02, Nanjing, China), and the cloning was performed according to the manufacturer’s instruction with a few modifications. In brief, the recombination reaction took place in a small volume of 5 μL for 5 min using 1 μL of un-purified PCR product, followed by cooling down on ice for 2 min for subsequent transformation.

**Transformation and quick transformation**

Briefly, the DNA for transformation was mixed gently with the thawed competent *E. coli*, followed by incubation on ice for 30 min. Subsequently, the mixture was heat-shocked for 60 s at 42°C. After another 10 min on ice, the transformed bacteria were recovered in SOC medium without antibiotics at 37°C for 1 h, which was plated on an agar plate supplemented with ampicillin and incubated overnight at 37°C. In the quick transformation protocol, the bacteria-DNA mixture was incubated on ice for 2 min, followed by heat shock for 60 s at 42°C, and then was directly plated to agar plates immediately after being cooled down on ice for 2 min. Abbreviations: SOC, Super Optimal broth with Catabolite repression.

**Plasmid construction and sequencing**

Lentiviral vectors used in this study were all based on the pLKO.1-puro vector backbone (Plasmid #8453, Addgene, MA, USA). The SuperSH method was depicted in Supplementary Figure S1a. Briefly, a pair of designed primers was used to amplify the vector backbone from a linearized template that was generated by digesting the intermediate vector pSuperSH-MX using *MluI* and *XbaI*, the product of which was subjected to seamless cloning and a subsequent quick transformation. Two colonies were picked for each construct and confirmed by sequencing. To construct the intermediate vector pSuperSH-MX, we followed the protocol of the SuperSH method using the primer pair sh-MluI-XbaI-Fd (5'-ACGCGTTCTAGATTTTTGAATTCTCGACCTCGAG-3') and sh-XbaI-MluI-Rv (5'-AAATCTAGAACGCGTACCGGTGTTTCGTCCTTTCC-3'). To clone pLKO.miRN-MluI-XbaI vector for miR-N shRNA vector cloning, we amplified the microRNA30a-encoding region with a 135-bp flanking sequence on each side using primers miR30-Fd (5’-GAAAGGACGAAACACCATTGCTGTTTGAATGAGGCT-3’) and miR30-Rv (5’-TGTCTCGAGGTCGAGAATTCAAAAAGACATGGTTTTAAAGTGATT-3’), which was cloned into pLKO.1 vector by seamless cloning to generate pLKO.30a. Subsequently, another round of PCR was performed with primers miR-N-Fd (5’-TGCCTACTGCCTCGGAC-3’) and miR-N-Rv (5’-CCGAGGCAGTAGGCATCTAGAACGCGTGTCGCTCACTGTCAACAG-3’) to obtain pLKO.miRN-MluI-XbaI for miR-N shRNA vector cloning. The shRNA sequences targeting CD44 with 21-mer guide sequence were obtained from Sigma-Aldrich website (<http://www.sigmaaldrich.com/china-mainland/zh/life-science/functional-genomics-and-rnai/shrna/individual-genes.html>). Guide sequences for 29-mer shRNA were designed by extending the matched 21-mer counterparts. The shRNA sequences accommodated for miR-N backbone were accessible on the Internet (<https://felixfadams.shinyapps.io/miRN>). Primers used for shRNA vector cloning was listed in Supplementary Table S1. All plasmids were sequenced at Sangon Biotech. (Shanghai, China) using the sequencing primer 5'-TACGATACAAGGCTGTTAGAGAG-3'. Abbreviations: SuperSH, Super rapid cloning of shRNA vector; shRNA, short hairpin RNA.

**Western blotting**

Total cell protein was extracted by lysing cell pellets with RIPA lysis buffer (P0013B, Beyotime, Shanghai, China) supplemented with protease inhibitor cocktail (P1010, Beyotime, Shanghai, China), followed by quantifying with BCA kit (#23225, Thermo Fisher, MA, USA). An equal amount of total protein of each sample was separated by 10% SDS-PAGE. The following primary antibodies were used: anti-GAPDH (1:1000, HC301-02) and anti-β-tubulin (1:1000, HC101-01) from TransGen (Beijing, China); anti-CD44 (1:1000, #3570) and anti-vimentin (1:1000, #5741) from Cell Signaling Technology (MA, USA). The secondary antibodies conjugated with HRP, anti-mouse (1:5000; HS201-01) and anti-rabbit (1:5000, HS101-01), were purchased from TransGen (Beijing, China). Signaling was detected using Western chemiluminescence substrate (WBLUF0100, Millipore, MA, USA) on chemiluminescence apparatus (MiniChemi, SAGE, Beijing, China). Abbreviations: SDS-PAGE, Sodium Dodecyl Sulfate-Polyacrylamide Gel Electrophoresis; GAPDH, Glyceraldehyde-3-phosphate Dehydrogenase; HRP, Horseradish Peroxidase.

**MTT, colony formation, and transwell assay**

MTT assay was performed by seeding 400 cells into each well of 96-well plate with 3 replicates. OD490 was detected at 3, 5, and 7 days, with the color product dissolved in DMSO for 10 min at room temperature after incubation in the presence of 1 mg/mL MTT for 4 h at 37°C. To perform colony formation assay, 3000 cells were plated into each well of 6-well plates and cultured for at least one week until visible colonies appeared. The colonies were then fixed with 4% formaldehyde at room temperature for 15 min, followed by staining with 0.1% crystal violet dissolved in PBS for 1 h. In transwell assay, 50,000 cells were carefully dropped into upper wells coated with 15% Matrigel (354234, BD, NJ, USA) and incubated at 37°C for 36 h with medium devoid of FBS (Gibco, CA, USA), for which their respective lower wells were filled with complete medium. After 36 h, the cells in the matrigel were carefully wiped off and the upper wells were fixed with methanol-acetic acid mixture (3:1) for 30 min, followed by staining with 0.5% crystal violet for 30 min. Abbreviations: FBS, Fetal Bovine Serum; MTT assay: 3-(4,5-Dimethylthiazol-2-yl)-2,5-Diphenyltetrazolium Bromide assay; OD490, Optical Density at 490 nm; DMSO, Dimethyl Sulfoxide; PBS, Phosphate Buffer Saline.

**References:**

1. Barretina J, Caponigro G, Stransky N, Venkatesan K, Margolin AA, Kim S et al. The Cancer Cell Line Encyclopedia enables predictive modeling of anticancer drug sensitivity. Nature. 2012;483(7391):603-7. doi:10.1038/nature11003.

2. Neve RM, Chin K, Fridlyand J, Yeh J, Baehner FL, Fevr T et al. A collection of breast cancer cell lines for the study of functionally distinct cancer subtypes. Cancer cell. 2006;10(6):515-27. doi:10.1016/j.ccr.2006.10.008.
